# Assessment of the Effects of Roasting, Contact Grilling, Microwave Processing, and Steaming on the Functional Characteristics of Bell Pepper (*Capsicum annuum* L.)

**DOI:** 10.3390/molecules29010077

**Published:** 2023-12-22

**Authors:** Remigiusz Olędzki, Joanna Harasym

**Affiliations:** 1 Department of Biotechnology and Food Analysis, Wroclaw University of Economics and Business, Komandorska 118/120, 53-345 Wroclaw, Poland; 2 Adaptive Food Systems Accelerator-Science Centre, Wroclaw University of Economics and Business, Komandorska 118/120, 53-345 Wroclaw, Poland

**Keywords:** bell pepper, steaming, roasting, microwave, *Capsicum annuum*, polyphenols, color, texture

## Abstract

Bell peppers (*Capsicum annuum* L.) in various stages of maturity are widely used in the diets of individuals and in the food industry; they are consumed both fresh and after thermal processing. However, every type of processing impacts the overall textural and bioactive characteristics of this plant-based food. In order to quantify the changes in the bioactive substances and color-structural characteristics that occur during selected heat treatments (contact grilling, roasting, roasting combined with microwaving, and steam cooking) of bell peppers at three maturity stages (green, yellow, and red), analyses of antioxidant activity, reducing sugar content, polyphenolic compound content, textural properties, and color coordinates in the L*a*b* system were carried out. Some of the processes used, such as contact grilling (15.43 mg GAE/g d.b.) and roasting combined with microwaving (15.24 mg GAE/g d.b.), proved to be beneficial as the total polyphenol content of green peppers (2.75 mg GAE/g d.b.) increased. The roasting (3.49 mg TE/g d.b.) and steaming (6.45 mg TE/g d.b.) methods decreased the antioxidant activity of yellow bell peppers (14.29 mg TE/g d.b.). Meanwhile, the roasting (0.88 mg Glc/g d.b.), contact-grilling (2.19 mg Glc/g d.b.), simultaneous microwaving and roasting (0.66 mg Glc/g d.b.), and steaming (1.30 mg Glc/g d.b.) methods significantly reduced the content of reducing sugars and reducing substances in red bell peppers (4.41 mg Glc/g d.b.). The studies proved that in order to preserve the antioxidant and bioactive properties of bell peppers, it is necessary to consider the use of appropriately selected heat treatments, depending on the different stages of maturity. The proper selection of adequate thermal treatment can not only increase digestibility, but also improve the bioavailability of bioactive substances from this raw material.

## 1. Introduction

The bell pepper (*Capsicum annuum* L.) is a vegetable that is cultivated almost worldwide and is consumed both fresh and after thermal processing; it is also used as a type of spice [1]. The bell pepper, which belongs to the Solanaceae family, includes many species and varieties, both sweet and spicy [2]. Regardless of the cultivar, annual bell pepper fruits are a rich source of polyphenolic compounds, which can be found in the range of 9.2–15.4 mg GAE/100 g of fresh weight (f.w.) [3]. Polyphenols have been confirmed to have an important function in protecting the body from reactive oxygen species (ROS), which are one of the main initiators of many diseases [4]. The consumption of flavan-3-ols, for example, has been proved to be associated with a reduced risk of myocardial infarction, stroke, and diabetes [5].

Polyphenols present in the diet have also been shown to help modify the blood lipid profile, normalize blood pressure, reduce insulin resistance, and reduce systemic inflammation [6,7], while the consumption of polyphenols such as quercetin and resveratrol has been linked to improved cardiovascular function [8]. Analyses using high-performance liquid chromatography coupled with tandem mass spectrometry and electrospray ionization (HPLC-ITMS) techniques have enabled the identification of a number of polyphenolic compounds in *Capsicum annuum* fruits, such as caffeic acid, coumaric acid, coumaroylquinic acid, 3-*O*-caffeoylquinic acid, ferulic acid, sinapic acid, apigenin-*O*-hexoside, and quercetin-*O*-rhamnosyl-*O*-hexoside [9]. The health-promoting and therapeutic potential of polyphenols present in the diet is also closely linked to the functioning of the gut microbiome, which converts polyphenolic compounds into a form that is highly bioactive and bioavailable [10,11]. According to a 2007 study by Anand et al. [12], the bioavailability of, for example, hexahydroxycurcumin (HHC), a metabolite formed by the biotransformation of curcumin, is significantly higher than the unmodified form of curcumin. 

Some researchers reported that the consumption of bell peppers (especially in the presence of fats) is beneficial due to their high content of carotenoids, which are essential for the differentiation process of human epithelial cells [13]. It has been confirmed that bell pepper fruits contain high amounts of carotenoids reaching levels, depending on the cultivar, from 133.9 mg/100 g to 324.2 mg/100 g dry basis (d.b.) [13,14]. The content of such carotenoids as lutein, zeaxanthin, α-carotene, and β-carotene in annual bell pepper fruits ranges from 1.95 to 3.12, 0.08 to 4.6, 0.0 to 5.16, and 0.0 to 44.42 µg/g d.b., respectively [15]. The activity and impact of carotenoids on the human system are related to the neutralization of free oxygen radicals, mainly singlet oxygen, so these substances help prevent or alleviate many chronic conditions and diseases, such as glandular cancer, diabetes, diseases of the visual system, cardiovascular diseases (such as ischemic heart disease), and aging processes [16].

Bell peppers also have a high content of ascorbic acid (vitamin C). Results show that the ascorbic acid content depends on the variety and maturity stage of the bell pepper, and can range from 16.52 to 107.3 mg/100 g of f.w. for green bell peppers, from 129.6 to 159.61 mg/100 g of f.w. for yellow bell peppers, and from 81.19 to 154.3 mg/100 g of f.w. for red bell peppers, respectively [17,18]. Ascorbic acid is a potent antioxidant, a cofactor for many biosynthetic enzymes, and for enzymes regulating the expression of genetic material [19,20]. In addition, ascorbic acid, of which annual bell peppers are a rich source, also strengthens immune defenses by supporting the cellular functions of both the innate and acquired immune systems [21]. This is because, among other things, phagocytic cells (such as neutrophils) have the ability to accumulate ascorbic acid, which enhances chemotaxis, phagocytosis, and the production of the reactive oxygen species needed by these cells to kill microorganisms [22]. For this reason, the consumption of raw materials rich in ascorbic acid results in increased immunity and a lower susceptibility to infection. Studies indicate that the consumption of ascorbic acid-rich raw materials or vitamin C supplementation (at 100–200 mg/day) helps to prevent respiratory tract infections and shortens the duration of systemic infections [23].

Therefore, due to the valuable bioactive and health-promoting properties of bell peppers, this raw material is often consumed in many types of dishes. Thus, the raw material is subjected to a different thermal-processing techniques that are standard in the food industry, such as roasting, contact grilling, steam cooking, or microwave processing. However, the use of these techniques is associated with changes in the bioactive properties and antioxidant content of the processed samples, which alter its health-promoting characteristics [24]. There is still a lack of studies explaining in detail the possible impact of various conventional thermal-processing methods on the content of the bioactive substances and, consequently, on their functional and health-promoting quality. 

The purpose of this article is to evaluate the effects of selected heat-treatment methods on the antioxidant properties and content of the bioactive substances of annual bell pepper fruits at different stages of maturity.

## 2. Results and Discussion

### 2.1. Total Polyphenols Content

The analysis of the raw materials showed that red bell peppers have the highest content of polyphenolic compounds, while green bell peppers have the lowest (Table 1). Similar results were obtained in Zhang et al.’s 2003 study [3], where it was confirmed that the total content of polyphenols in methanol extracts of green, yellow, and red bell peppers was 48.4, 54.8, and 64.5 mg GAE/100 g, respectively.

The results confirm that red bell peppers may be a raw material with high health-promoting potential. It has been found that consuming red bell peppers is related to a reduction in the risk of developing metabolic syndrome and the risk of death from cardiovascular disease, among other risks [25]. It has been shown that all the thermal treatments (R, MWR, CG, S) caused a statistically significant increase in the total content of polyphenolic compounds in green bell peppers. The greatest increase in the content of polyphenolic compounds was observed for green bell peppers after contact grilling (increase from 2.75 to 15.43 mg GAE/g d.b.).

On the other hand, in green bell pepper samples processed with simultaneous microwaving and roasting, a significant, more than five-fold increase, in the content of polyphenolic compounds (from 2.75 to 15.24 mg GAE/g d.b.) was observed, while a significant, more than two-fold increase (from 2.75 to 6.25 mg GAE/g d.b.) was observed as a result of steam cooking. Similar results were reported in our previous study for green bell peppers of the ‘Ożarowska’ cultivar (from the 2022 crop season);using a microwave treatment, it was observed that the total content of polyphenolic compounds was increased by more than 2.7 times (7.40 GAE µM/g d.b). Further, by cooking the peppers in boiling water, the total content of polyphenolic compounds was increased by more than 3.5 times (9.76 GAE µM/g d.b.) (compared to the raw material content of 2.75 GAE µM/g d.b.) [26].

Different results were obtained in a study by Özgür et al. in 2011 [27], where the total content of polyphenolic compounds in green bell peppers was reduced from 96.04 to 55.47 mg GAE/g during conventional oven drying, and in a study by Hameed et al. in 2023 [28], where steamed fresh green broccoli (*Brassica oleracea*) showed a higher total content of polyphenolic compounds (191.75 mg/g d.b.) compared to samples after microwave treatments (180.03 mg/g d.b.) and a hot-air-drying process in an oven (176.85 mg/g d.b.) [28].

It was also found that all the heat-treatment methods used (R, MWR, CG, S) caused a reduction in the total content of polyphenolic compounds in yellow bell peppers. The greatest reduction was observed for yellow bell peppers that underwent a steam-cooking treatment (from17.00 to 6.31 mg GAE/g d.b). In contrast, oven treatment resulted only in a 47% reduction in total polyphenolic compounds (from 17.00 to 9.02 mg GAE/g d.b.).

Similar results were reported in an earlier study of yellow bell peppers of the ‘Ożarowska’ cultivar, where a significant reduction of more than 40% in the total content of polyphenolic compounds was observed as a result of microwave treatment (10.14 GAE µM/g d.b.) and cooking in boiling water (10.03 GAE µM/g d.b.) compared to the raw material (17.00 GAE µM/g d.b.) [26]. Also, a study by Reis et al. in 2013 [29] showed that the total content of polyphenolic compounds in yellow bell peppers of the ‘Cumari-do-Para’ bell pepper (Capsicum chinense) cultivar grown in Brazil was reduced due to drying (at 65 °C) from 9748.22 to 1415.44 mg GAE/kg.

For red bell peppers, a significant, more than three-fold reduction in the content of polyphenolic compounds was observed as a result of simultaneous microwaving and roasting (from17.26 to 5.46 mg GAE/g d.b.), as well as steam cooking (reduction from 17.26 to 6.01 mg GAE/g d.b.). Oppositely, grilling resulted in a significant 36% increase in the TPC of red bell peppers, from 17.26 to 23.56 mg GAE/g d.b. Similar results were recorded in an earlier study on red bell peppers, where a significant 11% reduction in the TPC was also observed as a result of microwave treatment (15.37 GAE µM/g d. b) as well as a significant more than nine-fold reduction in the TPC (1.86 GAE µM/g d.b.) as a result of boiling water cooking [26].

Different results were obtained in a study of red bell peppers of the ‘Capia’ bell pepper cultivar, which were subjected to conventional and microwave drying. Here, the TPC decreased from 1297.49 mg GAE/100 g to 392.95 mg GAE/100 g (by 69.71% for drying in a 720 W microwave oven for 16 min) and 348.64 mg GAE/100 g (by 73.13% for drying in a conventional oven at 120 °C for 100 min), respectively. On the other hand, a 69.05% reduction in the TPC of ‘Capia’ bell peppers to 401.54 mg GAE/100 g was observed when using a warm-air-drying process (drying process carried out in the open air for 1 day) [30]. Also, Özgür confirmed that the oven-drying process for cooking red bell peppers causes a reduction in total polyphenol content from 130.79 to 89.82 mg GAE/g [27].

Different results were obtained by Speranza et al. in 2019 [31] for red bell peppers (‘Senise’ cultivar) dried in an oven (with forced air circulation) for 48 h at 55 °C (and at an internal relative humidity of the air stream of about 15–17%). As a result of this experiment, it was shown that the drying process did not cause significant changes in the total content of polyphenolic compounds in the red bell peppers, which were grown in the Battipaglia region of southern Italy. In contrast, the same ‘Senise’ cultivar of bell peppers grown in the Montanaso region (northern Italy) had a 7.6% reduction in the total polyphenolic compounds after a heat-treatment process in a forced-air oven [31].

The microwave and roasting method (reduction from17.26 to 5.46 mg GAE/g d.b.), as well as the steaming method, resulted in a significant reduction in the total content of polyphenolic compounds in red bell peppers (reduction from 17.26 to 6.01 mg GAE/g d.b.). Our study showed that the contact-grilling method preserves more polyphenolic components than the microwave treatment combined with roasting method as well as the steam-cooking method. This can be attributed to more controlled heating conditions. In addition, steam cooking appears to cause the leaching of water-soluble antioxidants, which increases with the duration of the process. Polyphenols are substances that are mostly water-soluble. During the steaming process, polyphenol molecules may move into the particles of steam rising in the steamer. Since a significant decomposition of plant tissues occurs during heat treatment, there may be an intensive migration of cellular components (including polyphenols) from the bell pepper skin into the hot steam.

Arfaoui reported results that are highly consistent with our results about different heat-treatment methods, which proves that cooking causes the most drastic changes in the composition of polyphenolic compounds in processed vegetables [32]. For this reason, perhaps processes such as roasting, contact grilling, and microwaving combined with roasting resulted in a smaller reduction in the content of polyphenolic compounds than the steam-cooking process.

On the other hand, processes such as roasting and contact grilling may contribute to the degradation of polysaccharides such as hemicellulose and pectin, which fill the gaps between cellulose microfibrils in the cell wall of bell pepper cells [33]. This phenomenon leads to a shrinking of the cell wall, which may result in a retention of a large number of polyphenolic compounds inside the cells in the outer layers of the bell pepper skin.

At the same time, the observed changes may have been responsible for the decrease in the hardness parameter of all types of processed bell peppers (green, yellow, and red) as a result of the heat-treatment methods used.

A study by Zahoor et al. in 2023 [34] showed that the total polyphenol content of red bell peppers was significantly reduced from 86.39 to 75.10 mg GAE/100 g d.b. as a result of microwave treatment (with a microwave power of 320 W) at 50 °C. However, when the same process was carried out (at a microwave power of 320 W) at 70 °C, no significant reduction in the total content of polyphenolic compounds was observed, which was confirmed to be 85.23 mg GAE/100 g d.b. On the other hand, when the drying process was carried out at a microwave power of 480 W and at 70 °C, a significant increase in the total content of polyphenolic compounds was observed to 92.30 mg GAE/100 g d.b. [34].

In our study, heat treatment in the form of roasting had a beneficial effect on the total content of polyphenolic compounds in green and red bell peppers. In addition, in the case of yellow bell peppers, only a 10% reduction in the total content of polyphenolic compounds was observed as a result of the simultaneous roasting and microwaving process. The reason for the observed changes may be the release (under high temperature and microwave radiation) of polyphenolic compounds, which are strongly bound in the cell-wall matrix of unprocessed raw materials [35].

Presumably, the high temperature and microwave radiation(for the Y-MWR variant) caused the inactivation of polyphenol oxidase (PPO) and other oxidative enzymes, such as lipoxygenase, which were released from the damaged bell pepper cells [36]. This phenomenon may have significantly reduced the degradation of polyphenolic compounds in the yellow bell peppers due to the simultaneous roasting and microwaving process.

A partial confirmation of the results we obtained are found in the results of a study on the microwave treatment of black peppers (*Piper nigrum* L.), the treatment of which (lasting 15 min at a power of 300 W) caused a significant reduction (from 79.62 to 36.20 mg GAE/100 g d.b.) in the total polyphenol content [37]. Also, in a study by Kaur et al. in 2005 [38], it was shown that the total polyphenol content of yellow sweet ‘Bachata’ bell peppers (*Capsicum annuum* L.) that were subjected to hot-air convection drying (at three different temperatures of 40, 50, and 60 °C) was reduced from 784.01 mg GAE/100 g d.b. to 630.76 mg GAE/100 g d.b.

### 2.2. Total Antioxidant Activity vs. DPPH Radical

The analysis of the total antioxidant potential of raw bell peppers showed that red bell peppers had the highest antioxidant activity (20.00 mg TE/g d.b.), followed by yellow bell peppers (14.29 mg TE/g d.b.) and green bell peppers (11.77 mg TE/g d.b.).

Our results are confirmed by research conducted by Sun et al. in 2007 [39], where green bell peppers were shown to have an antioxidant activity of 2.1 μmol TE/g f.w., while red bell peppers had an antioxidant activity of 3.9 μmol TE/g f.w. In the current study, it was observed that all heat treatments reduced the total antioxidant activity (measured using the DPPH method) in green, yellow, and red bell peppers. The greatest reduction in antioxidant activity in green bell peppers occurred as a result of roasting treatments (Table 1).

The current study showed that the steam cooking of green, yellow, as well as red bell peppers preserves more antioxidant components than roasting and the combined microwave and roasting treatment. This can be attributed to more destructive heating conditions (than during steamer heating), which cause the degradation of thermolabile antioxidants like ascorbic acid [40].

Largely similar results were reported in earlier studies on green and yellow bell peppers of the ‘Ożarowska’ cultivar, where a significant 62.76% reduction in the total antioxidant activity due to microwave treatment was observed in green bell peppers (reduction from 74.91 to 47.02 TE µM/g d.b.) and a significant 43.83% reduction in the antioxidant activity in yellow bell peppers (reduction from 57.10 to 25.03 TE µM/g d.b.), relative to the raw material [26]. Also, in a study conducted by Hameed et al. in 2023 [28], it was shown that a steam treatment of fresh green broccoli (Brassica oleracea) preserves higher antioxidant activity (58.80 DPPH (%)) (expressed as a percentage of DPPH radical neutralization) than a microwave treatment (54.95 DPPH (%)). However, in the same experiment, a hot-air treatment (oven roasting) (expressed as a percentage of neutralized DPPH radicals) proved almost as beneficial as the steam treatment and gave the tested broccoli an antioxidant activity of 58.36 DPPH (%) [28].

The greatest reduction in antioxidant activity in yellow (reduction from 14.29 to 1.94 mg TE/g d.b.) and red (reduction from 20.00 to 1.27 mg TE/g d.b.) bell peppers was due to the combined microwaving and roasting treatment process. It was shown that the process of roasting red bell peppers reduced the antioxidant activity of the sample by as much as 97% relative to the raw material.

The intense red color of red bell pepper fruits is due to the presence of carotenoids, the biosynthesis of which occurs mainly during the ripening of the peppers [41]. Our results indicate that there is probably a greater loss of carotenoids due to roasting than during processing with other heat-treatment techniques. At the same time, contact grilling allows for the better retention of carotenoids (greater stability of bell peppers to heat treatment).

Similar results were obtained in a study of red bell peppers of the ‘Capia’ cultivar, where it was observed that the total antioxidant activity of bell peppers is reduced by drying in a conventional oven (at 120 °C), where there was a reduction in the antioxidant activity to 3.80 mmol TE/kg, from a level of 35.85 mmol TE/kg for the raw material. On the other hand, when a microwave oven-drying process was used (at 720 W for 16 min), a reduction in the antioxidant activity to 3.71 mmol TE/kg f.w. was observed. When drying in fresh and warm air (for 1 day) was applied, a reduction in the total antioxidant activity was observed in ‘Capia’ bell peppers to a level of 3.94 mmol TE/kg f.w. [30]. Substantially similar results were obtained in a study of red bell pepper fruits of the ‘Senise’ cultivar grown in the Battipaglia region (southern Italy) and Montanaso region (northern Italy). It was confirmed that the process of drying (roasting) in a forced-air oven caused a significant reduction in the total antioxidant activity measured using the DPPH method by 33 percent [30]. Another study showed that the antioxidant activity of fresh red bell peppers is reduced using conventional oven-drying (from 8720.70 to 694.55 μmol TE/g d.b.) [27].

Desai, in a study of black pepper (*Piper nigrum* L.) fruit, observed a significant reduction in the total antioxidant activity (expressed as a percentage of the DPPH radical inhibition from 85.12 to 53.97%) (measured using the DPPH method) when the raw material was microwaved (for 15 min at an appliance power of 300 W) [37].

Different results were obtained by Zahoor et al. in 2023 [34], where it was shown that the microwave-treatment process performed at a temperature of 60 °C resulted in a 60% increase in the antioxidant activity (expressed as the amount of extract required to neutralize 50% of the DPPH radicals) relative to the raw material.

### 2.3. Total Antioxidant Activity vs. ABTS Radical

Slightly different results were provided by the analysis of antioxidant activity using the ABTS method. The contact grilling (increase from 0.68 to 3.32 mg TE/g d.b.), simultaneous microwaving and grilling (increase from 0.68 to 1.05 mg TE/g d.b.), and steam cooking (increase from 0.68 to 3.79 mg TE/g d.b.) of green bell peppers were shown to significantly increase the antioxidant activity of the raw material.

A 2023 study by Hameed et al. [28] similarly showed that fresh green broccoli (Brassica oleracea), after steam treatment, exhibits a higher antioxidant activity (64.28 DPPH (%)) (expressed as percentage of the DPPH radical neutralization) than after microwave treatment (57.60 DPPH (%)). In the same experiment, hot-air treatment (baking) proved significantly less favorable than steam treatment and conferred antiradical activity on the tested broccoli (expressed as a percentage of the neutralized ABTS cation radicals at 63.69 ABTS (%)) [28].

On the other hand, in the case of yellow bell peppers, it was observed that contact grilling (reduction from 5.03 to 4.42 mg TE/g d.b.), roasting (reduction from 5.03 to 3.77 mg TE/g d.b.) and steaming (reduction from 5.03 to 4.03 mg TE/g d.b.) resulted in a significant reduction in the antioxidant activity as measured using the ABTS method. At the same time, it was shown that simultaneous microwaving and roasting processes (increase from 5.03 to 5.40 mg TE/g d.b.) result in a significant increase in antioxidant activity in yellow bell peppers. It was shown that all the heat-treatment processes used caused a significant reduction in the antioxidant activity of red bell peppers. The greatest reduction in the antioxidant potential of red bell peppers was observed due to roasting (reduction from 5.28 to 1.77 mg TE/g d.b.). In a study conducted by Desai, it was shown that there is a significant reduction (from 4.81 to 2.33 mmol TE/100 g d.b.) in the total antioxidant activity (measured using the ABTS method) in black pepper (*Piper nigrum* L.) fruits that were microwave-treated (lasting 15 min at a device power of 300 W) [37].

In contrast, the smallest reduction in antioxidant activity was caused by the contact grilling of red bell peppers (reduction from 5.28 to 4.34 mg TE/g d.b.). Perhaps this was due to the fact that contact grilling does not deprive the processed samples of antioxidants to the same extent as other processing methods [42].

### 2.4. Total Reducing Activity

It was shown that for all the samples, each of the thermal-processing methods used resulted in a significant reduction in the total oxidoreductive activity. In the case of green bell peppers, the greatest reduction in oxidoreductive activity was observed in the case of the combined microwaving and roasting process(from 10.54 to 0.76 μM FeSO_4_/g d.b.). In the case of green bell peppers, the process of roasting and steam-cooking resulted in the smallest reduction in oxidoreductive activity (from 10.54 to 1.44 μM FeSO_4_/g d.b.). Similarly, for yellow bell peppers, the roasting process caused the greatest reduction in oxidoreductive activity (from 19.93 to 0.82 μM FeSO_4_/g d.b.). In the case of yellow bell peppers, the contact-grilling process caused the smallest reduction in oxidoreductive activity compared to other heat treatments. However, it should be noted that in the case of yellow and red bell peppers, all heat-treatment processes caused a reduction in the oxidoreductive activity, with a similar degree of attenuation of the value of this parameter. In the case of red bell peppers, all heat-treatment processes caused a reduction in the oxidoreductive activity. In the case of red peppers, it was the roasting process that caused the greatest reduction in oxidoreductive activity (from 39.79 to 1.13 μM FeSO_4_/g d.b.). In contrast, for red bell peppers, the oxidoreductive activity was reduced to the least extent with contact grilling (from 39.79 to 1.62 μM FeSO_4_/g d.b.), as shown in Table 1.

Partially concurrent results were obtained in a study by Fong-in, where it was found that fresh edible Astraeus odoratus mushrooms (665 mg ascorbic acid/100 g d.b.) lose oxidoreductive potential as a result of steam cooking (510 mg ascorbic acid/100 g d.b.) and microwave treatment (445 mg ascorbic acid/100 g d.b.). The same study showed that the baking process (795 mg ascorbic acid/100 g d.b.), on the other hand, increases the oxidoreductive potential relative to the raw material (665 mg ascorbic acid/100 g d.b.). In contrast, the traditional grilling process (675 mg ascorbic acid/100 g d.b.) caused no change in the oxidoreductive potential of the tested raw material [43].

All thermal-treatment processes, such as roasting, contact grilling, steaming, and simultaneous roasting and microwaving, resulted in a reduction in the reducing activity for all types of tested bell peppers. Additionally, our study did not show significant differences in the reducing activity for all tested samples between the roasting process and the steaming process.

Reductive activity is closely related to the ability of a substance to donate electrons, which is revealed by the reduction of Fe^3+^ iron ions to Fe^2+^ iron ions. Lower values of reducing activity in the case of the thermal-treatment techniques used may result from the penetration of polyphenolic compounds into the water fraction (water vapor), or their flow out of the raw material together with the cell juice during roasting or contact grilling. Additionally, during thermal treatments, polyphenolic compounds can form strong hydrogen bonds with the sulfur amino acids present in bell peppers (such as cysteine and methionine), thus losing the ability to donate electrons [44].

The same observed changes were probably accompanied by a loss of turgor in the bell pepper cells, which led to a decrease in the hardness and chewiness of all types of processed bell peppers (green, yellow, and red) as a result of the heat-treatment methods used. This, in turn, was observed as the processed samples became softer and less firm at the same time.

Slightly different results were obtained in research conducted by Nandasiri, where it was shown that baking using the air-fry technique (baking with hot air) allowed for the maintenance of a higher reducing activity of broccoli sprouts (measured using the FRAP method at the level of 0.37 mM TE/g d.b.), and Brussels sprouts (at the level of 0.26 mM TE/g d.b.), than during the steam-cooking process, where values of 0.13 mM TE/g d.b. were confirmed for these materials, 0.14 and 0.8 mM TE/g d.b., respectively.

Similarly, it was observed that the air-frying treatment allowed the maintenance of a higher reducing activity (measured using the FRAP method) of kale leaves (0.31 mM TE/g d.b.), red cabbage (0.265 mM TE/g d.b.), and green cabbage (0.16 mM TE/g d.b.). This was more beneficial than the steaming process for these raw materials, where a reduction activity was observed at levels of 0.15, 0.09, and 0.055 mM TE/g d.b., respectively [45].

### 2.5. Reducing Sugars Content

It was shown that all the heat-treatment methods used reduced the reducing sugars content in green, yellow, and red bell peppers. The greatest loss of reducing sugars in green bell peppers was observed from the simultaneous microwaving and grilling of this raw material (from 4.15 to 1.88 mg Glc/g d.b.). In the case of yellow bell peppers, the greatest reduction in reducing sugars content was observed for bell peppers that were steam-treated (from 5.61 to 0.80 mg Glc/g d.b.). On the other hand, the greatest decrease in reducing sugars content was observed for red bell peppers that were steam-treated (from 4.41 to 0.88 mg Glc/g d.b.).

Similar results were obtained in a study of red bell peppers of the ‘Senise’ cultivar grown in the Montanaso region (northern Italy), which were subjected to roasting in a forced-air oven, resulting in a significant reduction of 28% in total reducing sugars [31]. Also, in a study conducted by Bianchi and Lo Scalzo in 2018 [46], it was observed that the drying process of hot chili bell pepper powder significantly reduces the content of sugars such as glucose and fructose. In a study that used a forced hot-air stream to dry Hungarian red sweet bell peppers (*Capsicum annuum*), a significant reduction in the reducing sugars content such as glucose and fructose was observed [47]. Perhaps the pronounced decrease in the content of reducing sugars in red bell peppers that we observed as a result of roasting was due to the utilization of reducing monosaccharides (mainly fructose), which underwent the Maillard reaction during the initial stages of thermal processing [48].

### 2.6. Color Profile Change after Treatments

Significant changes in the color of the analyzed samples were observed between the raw material and heat-treated samples. The color analysis showed that the values of L*, a*, and b* were significantly higher for roasted green peppers (GR), green peppers grilled using contact grilling (GCG), green bell peppers that were microwaved and roasted (GMWR) (except for the parameter a* for the GMWR sample), and green peppers that were processed using water steaming (GS) compared to the raw material (Table 2).

This indicates that processes such as roasting, simultaneous roasting and microwaving, and steam processing cause a loss of green color intensity (toward a brightening of the green color), while the contact-grilling process causes browning of the bell pepper surface.

Perhaps the particularly large increase in the b* value for green bell peppers due to the roasting process and the combined roasting and microwaving process was a result of the effects of reactive oxygen species, such as ozone, peroxides, and hydroxyl radicals, which oxidize the color substances contained in the skin of green bell peppers [49].

Reactive oxygen species that are generated during microwave processing cause the degradation of cell structures and the chlorophyll pigments they contain, such as chlorophyll a and b. Reactive oxygen species accelerate the degradation of chlorophyll during heat treatment to brown pheophytin, which is caused by the loss of the magnesium ion in the active center of the chlorophyll molecule [50].

On the other hand, in 2021 Darici et al. [51] reported that the microwave drying of green bell peppers (180 W) resulted in a decrease in L* and b* values, while a significant increase in a* values compared to fresh bell peppers was observed. This was indicated by the browning of the processed samples.

For green bell peppers, the smallest difference in color relative to the raw material was observed for the steam-treated bell peppers. After the combined microwave and roasting treatment, a delta E value of 11.4 was obtained for the green bell peppers (Table 3).

Similar results were obtained by Darıcı et al. in 2021 [51] (ΔE values of 13.97) for green bell peppers that were microwave-dried. On the other hand, in a study conducted by Eyarkai et al. in 2016 [52], it was shown that the blanching process for vegetables such as green peas, eggplant, and green bell peppers resulted in a reduction of all CIE color coordinates (L, a, b) in these raw materials, which was associated with a weakening of color as a result of the leaching and migration of color substances into hot water [53].

In the case of yellow bell peppers, on the other hand, it was observed that the heat-treatment methods used reduced the L* brightness value and the b* value. The a* value of yellow bell peppers was decreased in the case of the roasting process and the simultaneous roasting and microwaving process and increased due to contact grilling and steaming. The observed changes were related to the yellow bell peppers’ loss of its bright yellow color toward a green color due to roasting, as well as the loss of its intense bright yellow color toward a dark yellow color due to contact grilling. There was a loss of an intense bright yellow color due to the simultaneous microwaving and roasting method, and its transformation to a green–yellow color was observed. The smallest difference in color was observed for yellow bell peppers after the steam treatment, as shown in Table 3.

Darici reported the same results from a study on yellow sweet bell peppers of the ‘Charleston’ cultivar (bell peppers with an elongated conical shape) and yellow sweet bell peppers. The microwave drying of these raw materials (180 W) resulted in a decrease in L* and b* values, while a significant increase in a* values compared to fresh bell peppers was observed, which was indicated by a strong browning of the raw material [51].

It is possible that the heat-treatment techniques used resulted in increased browning (non-enzymatic browning) with the simultaneous decomposition of pigments present in yellow and red bell peppers, such as β-carotene, zeaxanthin, quercetin, and luteolin. The decomposition of these pigments may have reduced the L* value in the yellow and red bell peppers [54]. In addition, it was indicated that the drying phenomenon that accompanies such thermal treatments as roasting and microwaving can cause the oxidation of ascorbic acid to dehydroascorbic acid, which in turn leads to a decrease in the brightness of the surface of the processed samples [55,56].

In the case of red bell peppers, there was a reduction in the L*, a* and b* values due to the roasting, contact-grilling, and combined microwaving and roasting processes. This resulted in a brightening (loss of brightness) of the red color of peppers when roasted, a darkening of the color when contact-grilled, and a dulling of the surface of peppers that were both roasted and microwaved. Also, in another study on red bell peppers of the ‘Capia’ cultivar, a reduction in the L* (brightness) value was noted after air-drying this raw material (from 35.56 to 28.30). When using conventional oven drying (at 120 °C) and drying using a microwave oven (at 720 W for 16 min), a reduction in L* values was observed to 31.86 and 31.04, respectively [27]. The same study confirmed that the a* and b* values of red raw bell peppers (22.67), as a result of the heat-treatment methods used (oven-drying, microwave-drying, and air-drying processes) are significantly reduced [27]. Also, in a study conducted by Deng et al. in 2018 [57], it was shown that the L* values of red peppers after the drying process decreased from a value of 41.60 to 26.64.

However, in the case of red bell peppers, the steam treatment resulted in an increase in L* and b* values and a simultaneous decrease in the a* value. In the case of red bell peppers, steam cooking caused a marked brightening of the surface of the red bell peppers toward yellow (Table 3). Different results were obtained in a study conducted by Eyarkai et al. in 2016 [52], where the blanching process of beets caused a reduction in all CIE color coordinates (L*, a* and b*), which was associated with a weakening of the color intensity of this raw material.

Statistically significant differences were found in the C values between raw green bell peppers and roasted green bell peppers, contact-grilled green bell peppers, and green bell peppers that were both microwaved and roasted. There were also statistically significant differences in C values between raw yellow bell peppers and yellow bell peppers that were roasted, yellow bell peppers that were contact-grilled, yellow bell peppers that were processed with both microwaving and roasting, and yellow bell peppers that were treated with steam cooking. There were also statistically significant differences in C values between raw red bell peppers and roasted red peppers, red peppers grilled by the contact-grilling method, red bell peppers that were processed with both microwaving and roasting, and red bell peppers that were treated with steam cooking. There were statistically significant differences in h values between the raw material and the samples subjected to all heat treatments for all types of bell peppers tested.

### 2.7. Textural Profile Changes after Treatments

Changes in textural parameters depending on the type of heat-treatment method used are shown in Table 4.

Hardness is one of the most important textural parameters that change after heat treatment. Studies indicate that the hardness of the vegetable samples is strongly correlated with fiber content and is defined as the maximum value of the force measured at the first compression stage [58].

The results of the texture analysis showed that there was a significant reduction in the hardness of all types of processed peppers—green, yellow, and red—as a result of all four heat-treatment methods used (Table 4).

To the greatest extent, the analyzed raw materials were subjected to a reduction in hardness when they were subjected to contact grilling. Hardness reduction occurred by 93%, 69%, and 93%, respectively, for the raw green, yellow, and red bell peppers.

Similar results were obtained by Merve in a study of yellow sweet ‘Charleston’ peppers, green peppers, and yellow sweet bell peppers that were subjected to microwave drying, where a reduction in the hardness parameter was observed by 60%, 48%, and 14%, respectively, relative to the raw materials [51]. Studies indicate that heat-treatment processes such as microwave processing and roasting cause a strong reduction in the turgor potential (pressure) and solubilization of pectic substances, resulting in the collapse of cellular structures and the softening of highly hydrated tissues, such as those present in many varieties of annual peppers (*Capsicum annuum* L.) [59].

In our study, steam processing was shown to reduce the hardness of green, yellow, and red peppers by 84%, 64%, and 59%, respectively. Also, in a study conducted by Eyarkai et al. in 2016 [52], a significant reduction in the hardness of the vegetables studied was observed due to blanching. The greatest reduction in hardness (by 90%) was observed in green peppers, followed by eggplant (by 80%), green peas (by 60%), and a slight reduction (by 25%) in beets. Our study showed a significant reduction in the hardness of green, yellow, and red peppers due to roasting (roasting) by 88.65%, 64.82%, and 76.33%, respectively. Also, in a study on raw chickpea seeds (*Cicer arietinum* L.), it was shown that their hardness decreased significantly with an increase in roasting time [60].

No significant changes in cohesiveness were shown for green bell peppers as a result of all four heat treatments used. Similarly, no significant changes in cohesiveness were shown for yellow bell peppers as a result of the thermal-processing methods used, such as roasting, simultaneous microwaving and roasting, and contact grilling. Only as a result of steam processing was there a significant increase in consistency for yellow bell peppers relative to the raw material. There was also no significant change in consistency for red bell peppers as a result of the thermal treatments used, such as simultaneous microwaving and roasting, contact grilling, and steam processing. Only as a result of roasting was there a significant increase in consistency for red bell peppers relative to the raw material.

The significant increase in springiness was observed for green bell peppers as a result of roasting, contact-grilling, and steam-processing methods.

In contrast, no significant change in springiness was observed for yellow bell peppers as a result of all four heat treatments. For red bell peppers, a significant increase in chewiness (relative to the raw material) was observed only as a result of their processing with contact grilling. There was a significant reduction in the chewiness that was observed (relative to the raw material) in all processed types of samples—green, yellow, and red peppers—as a result of all four thermal-processing methods used. The reduction in chewiness occurred to the greatest extent for the contact grilling of green bell peppers, the simultaneous microwaving and roasting of yellow peppers, and the simultaneous microwaving and roasting of red bell peppers.

The results of the study showed that only the simultaneous microwaving and roasting method and steam-processing method significantly increased the resilience of green bell peppers. In contrast, no significant changes in resilience were observed for yellow and red peppers as a result of all four heat-treatment methods.

## 3. Materials and Methods

### 3.1. Analytical Reagents and Standards Used

The following analytical reagents were used for the analyses: glucose, Trolox (6-hydroxyl-2,5,7,8–tetramethylchromo-2-carboxylic acid, 2,2–diphenyl–1-picrylhydrazyl (DPPH), 2,2–azino–bis (3-ethyl benzothiazoline-6-sulphonic acid) (ABTS), TPTZ (2,3,5-triphenyltetrazolium chloride), gallic acid, and iron (III) sulphate hydrate (Pol-Aura, Zabrze, Poland). All solutions and buffers were prepared using distilled and deionized water.

### 3.2. Plant Research Material

The research material consisted of annual bell pepper fruits (*Capsicum annuum* L.), which were cultivated in the southern regions of Mazovia in Poland. The peppers were of the ‘Ożarowska’ cultivar. The bell pepper fruits were purchased in a fruit and vegetable store in Wrocław, Poland, in August 2022. The tested bell pepper fruits represented the three main stages of ripening (green, yellow, and red) of this cultivar. The fruits were stored for several dozen hours at a temperature of 4 °C until they were processed and analyzed.

### 3.3. Heating Procedures

The tested raw materials were pre-washed in running water and the bell pepper stalks were then removed by hand. Then, the raw materials were cut into fragments 7–8 cm long and about 4–5 cm wide, from which the seeds were removed. Then, the tested raw materials were subjected to four thermal treatments: roasting, roasting combined with microwaving, contact grilling, and steaming. The resulting samples are shown in Table 5.

#### 3.3.1. Steam Cooking

The washed fragments (quarters) of the bell peppers were drained of moisture and were then placed on a sieve in the upper part of a steamer (Clatronic DG 3547, Clatronic, Kempen, Germany) so that the raw material did not come into contact with the boiling water. The steaming time recommended for annual bell peppers (10 min) was counted from the moment the water boiled in the lower part of the device and produced hot steam, which generated a temperature of 110–120 °C inside the steamer.

#### 3.3.2. Contact Grilling

The washed fragments (quarters) of the bell peppers were drained of moisture and were then placed between the lower and upper grill plates (OptiGrill Elite XL GC760D electric grill with automatic programs, TEFAL, Rumilly, France). The recommended contact-roasting time for annual bell peppers (15 min) was counted from the moment the device heated up to a temperature of 200 °C.

#### 3.3.3. Roasting

The washed fragments (quarters) of the bell peppers were drained of moisture and were then placed in the oven chamber on the rotating tray of a multifunction microwave device (GE83X, Samsung Electronics, Suwon, Republic of Korea). The recommended microwaving time for annual bell peppers (15 min) was counted from the moment the device heated up to 180 °C.

#### 3.3.4. Simultaneous Roasting and Microwave

The washed fragments (quarters) of the bell peppers were drained of moisture and were then placed in the oven chamber on the rotating tray of a multifunction microwave device (GE83X, Samsung Electronics, Republic of Korea), which was thermally processed using a system of heaters and microwave radiation simultaneously. The recommended time for this type of processing (2 min and 30 s) was counted from the moment the device heated up to a temperature of 180 °C.

### 3.4. Extract Preparation

The raw materials and processed samples were homogenized using a manual homogenizer (CAT DI 18 Basic, Caterpillar, Irving, TX, USA). The resulting 5 g of homogenate was extracted with a 10 mL methanol and water (80/20 *v*/*v* %) mixture for one hour on a radial stirrer (MX-RD PRO, ChemLand, Stargard, Poland) at 60 rotations per minute, and then centrifuged (MPW-350, MPW, Warszawa, Poland) for 15 min. at 10,000 rpm and 5031 g. The supernatant obtained after centrifugation was analyzed for the total antioxidant potential, reduction potential, and content of bioactive substances.

### 3.5. Determination of Total Phenolic Compounds

The total content of polyphenolic compounds was determined spectrophotometrically (SEMCO, S91 E, Gdynia, Poland) using the Folin–Ciocalteu reagent according to the method of Yen et al. in 1995 [61] after introducing minor modifications. For this purpose, 0.1 mL of the Folin–Ciocalteu reagent and 1.58 mL of H_2_O were added to the obtained extracts (0.02 mL). After 5 min of incubation, 0.3 mL of a saturated sodium carbonate solution (Na_2_CO_3_) was added. The total phenolic compounds were determined after 20 min of incubation at 38 °C in the dark. As it reacts with polyphenolic substances, the Folin–Ciocalteu reagent forms a blue complex that has a maximum absorbance at 765 nm. A standard curve was prepared for gallic acid [61]. All the samples were analyzed in duplicate. The content of polyphenolic compounds in the tested material was calculated from the calibration curve and presented in milligrams of gallic acid equivalent (GEA) per gram of dry basis (dry weight)—d.b.

### 3.6. Determination of Antioxidant and Oxidoreductive Activities

#### 3.6.1. DPPH Test

The antioxidant capacity (in relation to stablethe 2,2-diphenyl-1-picrylhydrazyl radical (DPPH^•^)) for the raw materials and the processed samples was measured spectrophotometrically (SEMCO, S91 E, Poland) using the method of Klymenko et al. in 2019 [62] with minor modifications. For this purpose, 0.035 mL of the test solution was measured and added to 1 mL of (0.1 mM) a methanolic DPPH solution. The mixture was shaken and left at room temperature for 20 min, after which the absorbance was measured at 517 nm. All the samples were analyzed in duplicate [62]. The total antioxidant activity in the tested material was calculated from the calibration curve and presented in milligrams of Trolox equivalent (TE) per gram of dry basis (dry weight)—d.b.

#### 3.6.2. ABTS Test

The antiradical capacity of the raw materials and the processed samples against the cationic radical 2,2-azo-bis(3-ethylbenzothiazoline-6-sulfonic acid (ABTS^•+^) was measured spectrophotometrically (SEMCO, S91 E, Poland)using the Sridhar method (2019) with minor modifications. The ABTS^•+^ solution was prepared by mixing 7 mM of ABTS stock solution with 2.45 mM of potassium persulfate solution and incubating the mixture at room temperature (23 °C) in the dark for 16–24 h. The ABTS^•+^ solution was diluted with phosphate buffer (0.1 M) to give an absorbance of 0.900 ± 0.05 at 734 nm. To 1.0 mL of the diluted ABTS^•+^ solution, 0.02 mL of the extract to be tested was added. The absorbance at 734 nm was read exactly 10 s after mixing the test extract with the ABTS^•+^ solution. All the samples were analyzed in duplicate [63]. The total antioxidant activity in the tested material was calculated from the calibration curve and presented in milligrams of Trolox equivalent (TE) per 1 g of dry basis (dry weight)—d.b.

#### 3.6.3. FRAP Test

The reducing power (ability to reduce ferric ions Fe^3+^) of the raw materials and the processed samples was measured spectrophotometrically (SEMCO, S91 E, Poland) according to the method of Re et al. in 1999 [64], with minor modifications. The test extract was added to 1 mL of FRAP solution (acetate buffer (300 µM, pH 3.6), a solution of 10 µM TPTZ in 40 µM HCl and 20 µM FeCl_3_ in the ratio 10:1:1 (*v*/*v*)). The mixture was shaken and left at room temperature for 20 min, after which the absorbance was measured at 593 nm. All the samples were analyzed in duplicate [64]. The total reducing activity was calculated from the calibration curve and presented in milligrams of iron (II) sulfate equivalent FeSO_4_^−^·7H_2_O per 1 g dry basis (dry weight).

### 3.7. Measurement of Reducing Sugar Content

The sugar content of extracts for the raw materials and the processed samples was measured spectrophotometrically (SEMCO, S91 E, Poland) using a modified method according to Miller et al. in 1959 [65], taking advantage of the reducing properties of sugars towards 3,5-dinitrosalicylic acid (DNS). To 1 mL of the test sample, 1 mL of DNS reagent was added and mixed thoroughly. The resulting mixture was then heated in boiling water for 5 min. After the mixture cooled to room temperature, its absorbance at 535 nm was measured. All the samples were analyzed in duplicate [65]. The total content of reducing sugars in the tested material was presented in milligrams of glucose equivalent per gram of dry basis (dry weight).

### 3.8. Color Measurement

The color of the fresh and processed bell peppers’ surface was assessed using a Konica Minolta CR-310 chroma meter (Konica Minolta, Ramsey, NJ, USA) in CIE color space (L*,a*, b* co-ordinates). Color co-ordinates (L*, a*, b*), and parameters (chroma, hue) were taken in triplicate and expressed as means ± standard deviation [26].

### 3.9. Texture Measurements

The texture of the bell peppers was examined using a TPA test (Texture Profile Analysis) with an AXIS texture analyzer FC200STAV500 (AXIS, Gdansk, Poland) with the software AXIS FM v.2.18, as previously reported by Olędzki & Harasym [26].

In order to determine the textural properties of bell peppers, an analysis of the texture profile was carried out on cylindrical samples with a diameter of 20 mm and a 5 mm height, taken with the skin from the middle fragments of the fruit. Measurements to the fresh bell peppers were performed on the skin (external) side.

Hardness was the force expressed in the unit of newton (N) at the maximum deformation, whereas cohesiveness, springiness, chewiness, and resilience were calculated from two peaks, corresponding, respectively, to the first and second sample compression cycle. The analysis was carried out in quadruplicate at 25 °C on bell pepper cylinders cut from different pieces of each bell pepper fruit. The results were reported as means (from four measurements from each sample) ± standard deviation [26].

### 3.10. Dry Basis Assessment

Four samples were taken from each raw pepper and processed pepper. Each sample was cut into small pieces and 2 g of the chopped sample was placed on an aluminum plate in a weighing–drying machine (MA-30, Sartorius GmbH, Göttingen, Germany). An automatic method was set in the machine’s operating software, according to which the samples were dried at 105 °C. From the data obtained, the average of four measurements was calculated for each type of bell pepper. The obtained dry matter contents served as a reference point for presenting the results of the antioxidant activity, reducing activity, total content of polyphenolic compounds, and content of reducing sugars in the tested peppers.

### 3.11. Statistical Analysis

The results from all methods are given as mean values and their standard deviation. Data were subjected to one-way and two-way analysis of variance and mean values were compared using Tukey’s test (*p* < 0.05). Values of *p*< 0.05 were considered statistically significant. The statistical analysis was performed using Statgraphics Centurion 19 (Statgraphics Technologies, Inc., The Plains, VA, USA) statistical software.

## 4. Conclusions

The study showed that the heat-treatment methods used have significant and differential effects on the antioxidant properties and bioactive content of fresh bell pepper fruits. For green bell peppers, the thermal processes that preserve more antioxidant components were contact grilling and steaming.

For yellow bell peppers, on the other hand, the thermal processes that proved to be beneficial in terms of polyphenol content were the simultaneous microwave and roasting method and contact-grilling method. However, due to the antioxidant activity, processes such as contact grilling and steaming appeared to be beneficial for yellow bell peppers. For red bell peppers, roasting proved to be an exceptionally favorable thermal-treatment process, which even increased the total content of phenolic compounds (relative to the raw material). On the other hand, in terms of antioxidant activity, contact-grilling and steaming processes proved to be favorable processes for red bell peppers.

The proper selection of the thermal-treatment process according to the ripeness and cultivar of bell peppers can increase not only their digestibility, but also improve the bioavailability of nutrients and bioactive substances contained in the raw material. Therefore, to ensure the optimal intake of antioxidants contained in processed bell pepper fruits, it is necessary to consider the use (during food preparation) of separate heat-treatment processes for different stages of bell pepper maturity (bell pepper color forms). The obtained results suggest that in the future it would be good to develop industry guidelines for the food and catering industry regarding thermal-processing methods for vegetable and fruit plant raw materials.

## Figures and Tables

**Table 1:** The total polyphenol content, antioxidant activity, and reducing sugar content of bell peppers after different thermal-processing treatments.

R-Stage	Processing	SUGARS mg GlcE/g d.b.	TPC mg GAE/g d.b.	DPPH mg TE/g d.b.	ABTS mg TE/g d.b.	FRAP μM FeSO_4_/g d.b.
G	RM	4.15 ± 0.17 ^b^	2.75 ± 0.42 ^a^	11.77 ±0.37 ^c^	0.68 ± 0.16 ^a^	10.54 ± 0.37 ^c^
	CG	1.99 ± 0.16 ^a^	15.43 ± 1.15 ^c^	7.27 ± 0.52 ^b^	3.32 ± 0.09 ^c^	1.37 ± 0.07 ^b^
	R	2.27 ± 0.20 ^a^	7.09 ± 0.57 ^b^	1.57 ± 0.06 ^a^	0.58 ± 0.13 ^a^	1.43 ± 0.08 ^b^
	MWR	1.88 ± 0.13 ^a^	15.24 ± 0.01 ^c^	1.94 ± 0.13 ^a^	1.05 ± 0.10 ^b^	0.76 ± 0.04 ^a^
	S	2.07 ± 0.62 ^a^	6.25 ± 0.47 ^b^	7.18 ± 0.42 ^b^	3.79 ± 0.18 ^d^	1.44 ± 0.05 ^b^
Y	RM	5.61 ± 0.08 ^d^	17.00 ± 0.68 ^e^	14.29 ± 0.51 ^e^	5.03 ± 0.12 ^d^	19.93 ±1.64 ^b^
	CG	1.45 ± 0.21 ^c^	12.56 ± 0.68 ^c^	8.73 ± 0.77 ^d^	4.42 ± 0.11 ^c^	1.79 ± 0.13 ^a^
	R	1.12 ± 0.08 ^b^	9.02 ± 0.28 ^b^	3.49 ± 0.11 ^b^	3.77 ± 0.06 ^a^	0.82 ± 0.04 ^a^
	MWR	1.16 ± 0.06 ^b^	15.24 ± 0.35 ^d^	1.94 ± 0.16 ^a^	5.40 ± 0.04 ^e^	1.08 ± 0.04 ^a^
	S	0.80 ± 0.04 ^a^	6.31 ± 0.11 ^a^	6.45 ± 0.50 ^c^	4.03 ± 0.04 ^b^	1.17 ± 0.00 ^a^
R	RM	4.41 ± 0.12 ^d^	17.26 ± 0.64 ^b^	20.00 ± 0.51 ^c^	5.28 ± 0.10 ^d^	39.79 ± 1.05 ^b^
	CG	2.19 ± 0.35 ^c^	17.20 ± 2.57 ^b^	9.29 ± 0.81 ^c^	4.34 ± 0.04 ^c^	1.62 ± 0.08 ^a^
	R	0.88 ± 0.04 ^ab^	23.56 ± 0.35 ^c^	1.40 ± 0.14 ^a^	1.77 ± 0.06 ^a^	1.13 ± 0.04 ^a^
	MWR	0.66 ± 0.01 ^a^	5.46 ± 0.36 ^a^	1.27 ± 0.05 ^a^	2.82 ± 0.14 ^b^	1.29 ± 0.01 ^a^
	S	1.30 ± 0.26 ^b^	6.01 ± 0.69 ^a^	7.91 ± 0.55 ^b^	4.16 ± 0.18 ^c^	1.46 ± 0.20 ^a^
processing		***	***	***	***	***
R-stage		***	***	***	***	***
processing × R-stage		***	***	***	***	***

R-stage—ripening stage; G—green pepper; Y—yellow pepper; R—red pepper. For Processing: RM—raw material, CG—contact grilling, R—roasting, MWR—microwave plus roasting, S—steaming. TPC—total polyphenols content; GlcE—glucose equivalent; GAE—gallic acid equivalent; TE—Trolox equivalent. Lower-case letters in columns mean that a statistically significant difference was found between samples for same R-stage (*p* = 0.05), ***—*p* < 0.001 for interaction between factors.

**Table 2:** Color values of samples in the L*, a*, b* (CIELAB) color space.

R-Stage	Processing	L*	a*	b*	C	h
G	RM	54.88 ± 2.00 ^a^	−13.80 ± 0.46 ^b^	15.40 ± 0.63 ^a^	20.68± 0.76 ^b^	131.83 ± 0.50 ^e^
	CG	59.57 ± 0.07 ^b^	−3.23 ± 0.06 ^d^	17.51 ± 0.02 ^b^	17.81 ± 0.03 ^a^	100.40 ± 0.17 ^a^
	R	67.44 ± 0.40 ^d^	−11.11 ± 0.39 ^c^	28.61 ± 0.98 ^d^	30.69 ± 1.05 ^c^	111.13 ± 0.25 ^b^
	MWR	61.07 ± 0.23 ^b^	−16.14 ± 0.10 ^a^	24.70 ± 0.21 ^c^	29.50 ± 0.23 ^c^	123.10 ± 0.10 ^c^
	S	65.32 ± 1.58 c	−10.89 ± 0.66 ^c^	15.49 ± 1.37 ^a^	18.93 ± 1.50 ^a^	125.10 ± 0.69 ^d^
Y	RM	99.28 ± 0.49 ^e^	3.36 ± 0.18 ^c^	89.63 ± 0.84 ^e^	89.69 ± 0.83 ^e^	87.93 ± 0.15 ^c^
	CG	89.80 ± 0.68 c	5.33 ± 0.16 ^d^	72.89 ± 0.96 ^c^	73.08 ± 0.97 ^c^	85.87 ± 0.06 ^b^
	R	51.79 ± 0.42 ^a^	−13.34 ± 0.07 ^a^	14.04 ± 0.22 ^a^	19.36 ± 0.21 ^a^	133.47 ± 0.31 ^e^
	MWR	84.42 ± 1.15 ^b^	−0.65 ± 0.07 ^b^	65.94 ± 2.11 ^b^	65.94 ± 2.11 ^b^	90.53 ± 0.06 ^d^
	S	97.78 ± 0.12 ^d^	10.72 ± 0.10 ^e^	85.23 ± 1.16 ^d^	85.89 ± 1.17 ^d^	82.90 ± 0.00 ^a^
R	RM	64.25 ± 0.53 ^c^	54.82 ± 0.85 ^d^	36.65 ± 1.18 ^d^	65.94 ± 1.35 ^c^	33.70 ± 0.48 ^c^
	CG	57.52 ± 1.02 ^a^	47.68 ± 0.76 ^a^	27.82 ± 0.71 ^a^	55.20 ± 1.01 ^a^	30.20 ± 0.27 ^a^
	R	62.18 ± 0.76 ^b^	51.49 ± 1.84 ^c^	33.47 ± 1.64 ^c^	61.41 ± 2.43 ^b^	32.95 ± 0.37 ^b^
	MWR	62.66 ± 0.09 ^b^	45.20 ± 0.17 ^b^	29.95 ± 0.16 ^b^	54.22 ± 0.08 ^a^	33.45 ± 0.25 ^bc^
	S	95.11 ± 0.47 ^d^	2.90 ± 1.08 ^e^	80.12 ± 0.48 ^e^	80.18 ± 0.50 ^c^	88.00 ± 0.74 ^d^

R-stage—ripening stage; G—green pepper; Y—yellow pepper; R—red pepper. For Processing: RM—raw material; CG—contact grilling; R—roasting; MWR—microwave plus roasting; S—steaming. Lower-case letters means that a statistically significant difference was found between samples for the same R-stage.

**Table 3:** Delta E values for the tested bell peppers.

R-Stage	RM-R	RM-MVR	RM-S	RM-CG
G	19.0 ± 2.2	11.4 ± 0.7	10.3 ± 3.2	11.7 ± 0.9
Y	90.2 ± 1.0	28.1 ± 3.0	8.5 ± 0.6	18.2 ± 2.8
R	5.1 ± 4.0	11.8 ± 1.5	74.4 ± 0.6	13.2 ± 1.2

For R-stage: G—green pepper; Y—yellow pepper; R—red pepper. For Processing: RM—raw material; CG—contact grilling; R—roasting; MWR—microwave plus roasting; S—steaming.

**Table 4:** Textural properties of the tested bell peppers.

R-Stage	Processing	Hardness [N]	Cohesiveness	Springiness	Chewiness [N]	Resilience
G	RM	76.7 ± 0.1 ^e^	0.784 ± 0.023 ^ab^	0.667 ± 0.000 ^a^	40.01 ± 1.14 ^e^	0.68 ± 0.09 ^ab^
	CG	5.4 ± 0.1 ^a^	0.726 ± 0.028 ^a^	0.838 ± 0.028 ^b^	3.22 ± 0.18 ^a^	0.76 ± 0.11 ^bc^
	R	8.7 ± 0.4 ^b^	0.839 ± 0.065 ^b^	0.926 ± 0.004 ^b^	6.69 ± 0.19 ^c^	0.57 ± 0.02 ^a^
	MWR	10.4 ± 0.1 ^c^	0.755 ± 0.005 ^ab^	0.641 ± 0.037 ^a^	5.02 ± 0.17 ^b^	0.98 ± 0.06 ^d^
	S	12.2 ± 0.9 ^d^	0.841 ± 0.030 ^b^	1.046 ± 0.064 ^c^	10.63 ± 0.50 ^d^	0.87 ± 0.02 ^cd^
Y	RM	34.4 ± 2.9 ^b^	0.818 ± 0.057 ^a^	0.701 ± 0.018 ^a^	19.73 ± 2.49 ^b^	0.86 ± 0.18 ^a^
	CG	10.4 ± 0.4 ^a^	0.824 ± 0.036 ^ab^	0.834 ± 0.235 ^a^	7.11 ± 2.08 ^a^	0.73 ± 0.21 ^a^
	R	12.1 ± 1.0 ^a^	0.868 ± 0.009 ^ab^	0.727 ± 0.084 ^a^	7.64 ± 1.45 ^a^	0.68 ± 0.04 ^a^
	MWR	10.6 ± 0.4 ^a^	0.798 ± 0.018 ^a^	0.691 ± 0.033 ^a^	5.87 ± 0.63 ^a^	0.66 ± 0.02 a
	S	12.1 ± 1.2 ^a^	0.914 ± 0.036 ^b^	0.731 ± 0.272 ^a^	7.94 ± 2.50 ^a^	0.77 ± 0.09 ^a^
R	RM	48.6 ± 1.1^d^	0.720 ± 0.015 ^a^	0.622 ± 0.030 ^a^	21.73 ± 1.10 ^d^	0.73 ± 0.12 ^a^
	CG	3.3 ± 0.8 ^a^	0.698 ± 0.125 ^a^	1.084 ± 0.118 ^b^	2.56 ± 1.30 ^a^	0.67 ± 0.42 ^a^
	R	11.5 ± 0.1 ^b^	0.896 ± 0.008 ^b^	0.763 ± 0.100 ^ab^	7.86 ± 1.05 ^b^	0.70 ± 0.10 ^a^
	MWR	16.4 ± 2.9 ^c^	0.786 ± 0.013 ^ab^	0.818 ± 0.257 ^ab^	10.24 ± 1.64 ^bc^	0.74 ± 0.16 ^a^
	S	19.6 ± 0.6 ^c^	0.813 ± 0.035 ^ab^	0.718 ± 0.072 ^a^	11.42 ± 1.00 ^c^	0.88 ± 0.14 ^a^
processing		***	*	ns	***	ns
R-stage		***	***	*	***	ns
Processing × R-stage		***	ns	ns	***	ns

R-stage—ripening stage; G—green pepper; Y—yellow pepper; R—red pepper. For Processing: RM—raw material; CG—contact grilling; R—roasting; MWR—microwave plus roasting; S—steaming. Lower-case letters mean that a statistically significant difference was found between samples for the same R-stage. (*p* = 0.05), ns—statistically non-significant, *—*p* < 0.05, ***—*p* < 0.001.

**Table 5:** Bell pepper chunks before (raw material) and after each thermal treatment.

Processing	Green Bell Pepper	Yellow Bell Pepper	Red Bell Pepper
Raw Material—RM	*** 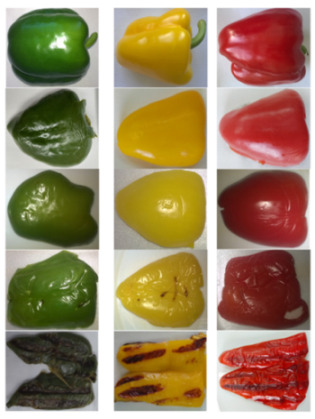 ***		
Steaming—S			
Roasting—R			
Roasting and Microwave—MWR			
Contact Grilling—CG			

## Data Availability

The data presented in this study are available on request from the corresponding author.
